# Supplementary material for: Pro-Resolving Mediators in Rotator Cuff Disease: How Is the Bursa Involved?
Source: Cells. 2023 Dec 20;13(1):17. doi: 10.3390/cells13010017 (PMC10778346; doi:10.3390/cells13010017)
Supplement: Supplementary file 1 [file cells-13-00017-s001.zip › cells-2739635-SI.pdf]

Supplementary Table S1: primer sequences in alphabetical order

Gene	Ascession Nb	Forward Primer	Reverse primer
**18s**	NM_022551	CGGAAATAGCCTTTGCCATC	AGTTCTCCCGCCCTCTTGGT
**ANXA1**	NM_000700	GAGATTTTCGGAACGCTTTG	CCCCTTTCTCCTTTCTCCTG
**ALOX5**	NM_000698.5	CTGGCTGAATGACGACTGGT	ACGTCGGTGTTGCTTGAGAA
**ALOX15**	NM_001140.4	GGGCAAGGAGACAGAACTCA	CAGCGGTAACAAGGGAACCT
**APOL3**	NM_145640.2	TGACTAACAATGAAGCCTGGAA	CCTCTTGACTTGGGGAAACTC
**ChemR23**	NM_004072	TCTGTCTTCAGCCTGGGTTT	ATGGCTGGGGTAGGAAGAGT
**CCL20**	NM_001130046.1	CTCCTGGCTGCTTTGATGTC	TGCGCACACAGACAACTTTT
**CD1D**	NM_001766.3	GGGTGAAGCACAGCAGTCTA	GGAGGTAAAGCCCACAATGA
**CD163**	NM_004244.5	TTTGTCAACTTGAGTCCCTTCAC	TCCCGCTACACTTGTTTTCAC
**CD206**	NM_002438	ACTGGGGCCAAGCTTCTCTG	CACAGCCACGTCCCTTCAAC
**CISH**	NM_013324.5	AGCCCAGACAGAGAGTGAGC	TGACAGCGTGAACAGGTAGC
**Col1A1**	NM_000088.3	TGACCTCAAGATGTGCCACT	ACCAGACATGCCTCTTGTCC
**Col3A1**	NM_000090.3	AGCCTGGTAAGAATGGTGCC	TCCTTGCCATCTTCGCCTTT
**CXCL11**	NM_005409.4	GACGCTGTCTTTGCATAGGC	TGGGATTTAGGCATCGTTGT
**FPR1** [26]	NM_001193306	TGGGAGGACATTGGCCTTTC	GGATGCAGGACGCAAACAC
**FPR2**	NM_001462	CTGAGGAGAGGCTGAAGGTG	GCAATGAGCCCATAGCAGAT
**FGL2**	NM_006682.2	AAAGTGTCCCAGCCAAGAAC	TCTCACTGCTTCTTTTGCCTA
**GPR18**	NM_001098200	CACCCCTCTGCTACTGCTCT	TGCCGTGAAGGAGATTATGA
**GPR32**	NM_001506.2	TTGCCAGTAACTGCCTCCTT	GCGTACAGCCATTCCATTTT
**HPRT**	NM_000194	GAAGGTGAAGGTCGGAGTC	GAAGATGGTGATGGGATTTC
**IL-1β** [34]	NM_000576	TCCAGGAGAATGACCTGAGC	GTGATCGTACAGGTGCATCG
**IL-6**	NM_000600	TGAGGAGACTTGCCTGGTGA	TTGGGTCAGGGGTGGTTATT
**IL-8**	NM_000584.3	TGGCAGCCTTCCTGATTTCT	TGGTCCACTCTCAATCACTCTCA
**IL-10**	NM_000572	TGAGAACAGCTGCACCCACT	GGCAACCCAGGTAACCCTTA
**IDO1**	NM_002164.5	TCACAGACCACAAGTCACAGC	TCCAGTTTGCCAAGACACAG
**IRF1**	NM_002198.2	CGATACAAAGCAGGGGAAAA	TTAGCATCTCGGCTGGACTT
**MMP-1**	NM_002421.3	CACGCCAGATTTGCCAAGAG	GTCCCGATGATCTCCCCTGA
**MMP-2**	NM_004530	TGGATGATGCCTTTGCTCGT	CCAGGAGTCCGTCCTTACCG
**Ppia**	NM_021130.4	TCTGAGCACTGGAGAGAAAGG	CAGGACCCGTATGCTTTAGG
**PTX3**	NM_002852.3	TTGCGATTCTGTTTTGTGCT	ATTCCGAGTGCTCCTGACC
**SRRM2**	NM_016333.3	CTCTGCTTCCTCCTCCGATA	GCCGTTGTGTGCTGGTAGTA
**TGM2**	NM_004613.3	AAGAGGAGCGGCAGGAGTAT	CAGGAACTTGGGGTTGACAT
**TIMP-1**	NM_003254.2	TTGGCTGTGAGGAATGCACA	AAGGTGACGGGACTGGAAGC
**TNF-α**	NM_000594	AGCCCATGTTGTAGCAAACC	GAGGTACAGGCCCTCTGATG
**VAMP5**	NM_006634.2	CAGCGTTCAGACCAACTCCT	CTGAGGGAGAAAGACGACCA
**WARS**	NM_004184.3	CATTTTCGGCTTCACTGACA	CATGGGATAAGGCACTGGAT

Supplementary Table S2: Protein level of release of pro-resolving and inflammatory mediators

pg/ml	Intact Rotator Cuff	Moderate Rotator Cuff Disease	Severe Rotator Cuff Disease	Kruskal Wallis Test
	Median (min-max)	Median (min-max)	Median (min-max)	p-value
**MCP-1**	5178.8 (593-25396)	12342.6 (250-24217)	11018.6 (20.2-38260)	0.509
**CXCL11**	181.0 (17.6-304)	211.4 (35.9-322)	229.9 (31.1-349)	0.334
**IFN-γ**	74.9 (17.5-152)	106.4 (22.5-184)	96.4 (8.3-218)	0.288
**IL-1β**	32.7 (10.1-72.8)	48.2 (0.4-83.1)	46.2 (10.1-454)	0.249
**IL-6**	2505.0 (58.5-10717)	4382.0 (18.7-10710)	3141.4 (0.3-10920)	0.702
**IL-8**	2276.6 (300-34793)	4556.7 (386-23963)	1843.3 (0.6-20511)	0.665
**IL-10**	29.7 (8.6-46.0)	36.9 (8.6-53.1)	37.5 (10.4-83.2)	0.259
**IL-13**	3531.8 (889-5201)	4127.7 (969-6453)	3336.9 (273-6200)	0.542
**IL-17**	9.1 (0.3-21.4)	12.2 (0.0-27.7)	10.6 (0.3-105)	0.413
**IL-1ra**	392.5 (31.9-1537)	843.1 (23.8-7966)	602.2 (0.0-28263)	0.251
**IL-6Rα**	61.4 (22.8-209)	99.5 (24.8-350)	126.1 (16.1-353)	0.188
**TNF-α**	14.6 (3.8-34.1)	22.1 (4.1-45.5)	19.0 (0.3-267)	0.332
**MMP-1**	5073.7 (715-66674)	9835.5 (556-92145)	12745.5 (0.4-592525)	0.289
**MMP-2**	20412.5 (3136-69311)	28627.1 (4747-93797)	23914.4 (1258-65657)	0.285
**MMP-3**	35407.1 (4839-275357)	105606.5 (10366-992867)	52880.5 (15.9-2668997)	0.260
**TIMP-1**	34072.8 (4677-53299)	41578.6 (4917-54142)	36413.3 (89.1-54911)	0.487
**Chemerin**	1846.1 (718-2706)	1856.2 (555-2508)	1886.3 (346-2317)	0.682

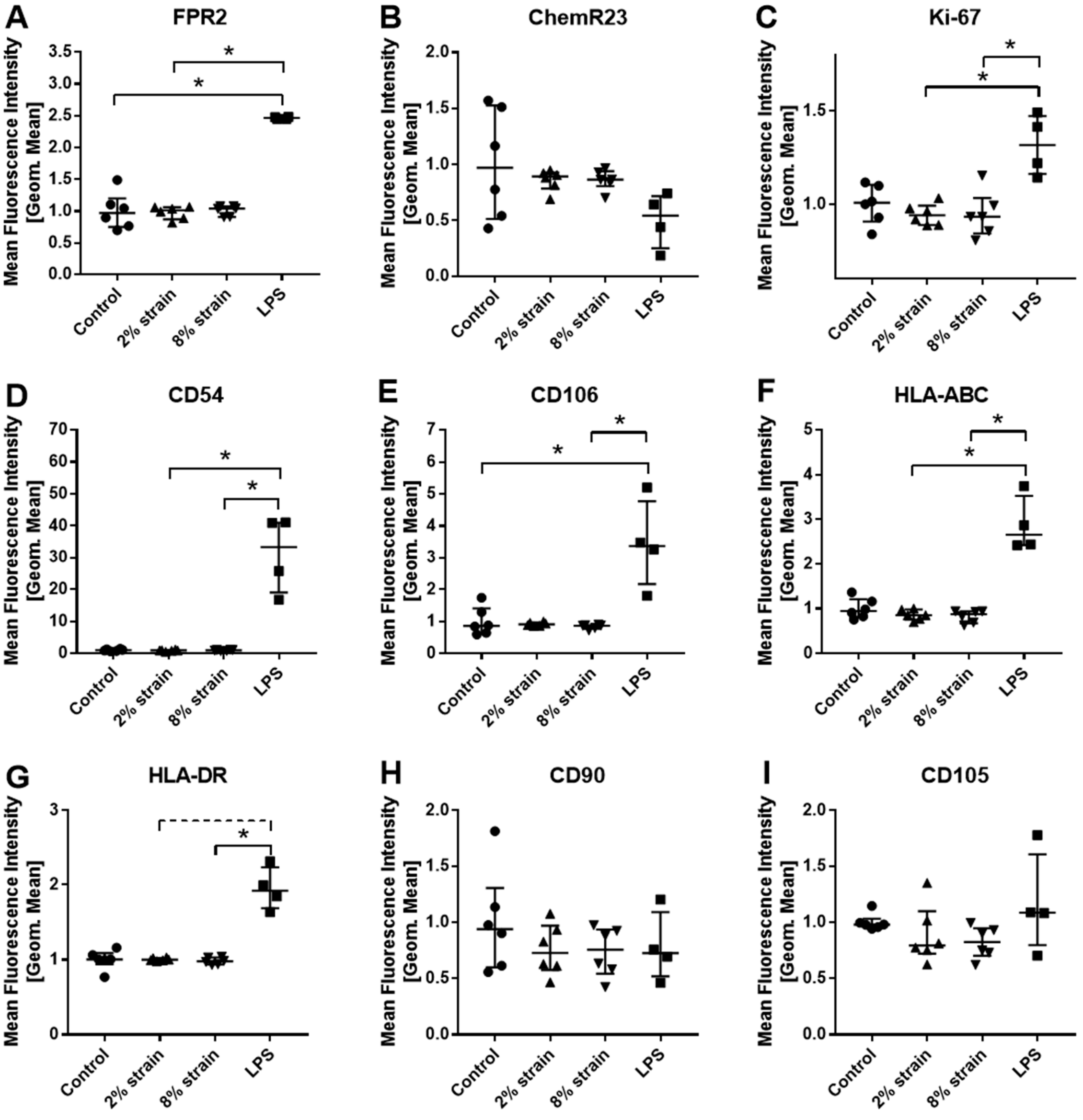

Supplementary Figure S1: Statistical evaluation of surface marker expression on bursa cells either left unstimulated (control, n=6), mechanically stimulated with 2% or 8% cyclic loading (each n=6) or stimulation with 100 ng/mL LPS (n=4). Surface marker expression is given as mean fluorescence (Geom. Mean) intensity normalized to the unstimulated control. Data are depicted as median with interquartile range. Statistics: Dunn´s Multiple Comparison test, * $p<0.05$, a dashed line indicate trends ($p<0.1$).
